# Adapting a Preparatory Skills‐Building Programme for Carers of People With Cancer Through Co‐Design: The iCanSupport Project

**DOI:** 10.1111/hex.70061

**Published:** 2024-10-17

**Authors:** Bróna Nic Giolla Easpaig, Bronwyn Newman, Judith Johnson, Ursula M. Sansom‐Daly, Lucy Jones, Lukas Hofstätter, Eden G. Robertson, Reema Harrison

**Affiliations:** ^1^ Australian Institute for Health Innovation Macquarie University Sydney New South Wales Australia; ^2^ School of Nursing, Faculty of Health Charles Darwin University Sydney New South Wales Australia; ^3^ Division of Nursing, Midwifery and Social Work, School of Health Sciences University of Manchester Manchester UK; ^4^ School of Clinical Medicine, Discipline of Paediatrics & Child Health, UNSW Medicine and Health, Randwick Clinical Campus UNSW Sydney Sydney New South Wales Australia; ^5^ Kids Cancer Centre, Sydney Children's Hospital Sydney New South Wales Australia; ^6^ Sydney Youth Cancer Service Nelune Comprehensive Cancer Centre, Prince of Wales Hospital Randwick New South Wales Australia; ^7^ Neuroblastoma Australia Sydney New South Wales Australia; ^8^ Carers NSW Sydney New South Wales Australia; ^9^ Redkite Sydney New South Wales Australia

**Keywords:** cancer, carers, co‐design, psychosocial intervention

## Abstract

**Introduction:**

Health services rely upon carers to provide care for loved ones with cancer, yet many carers often feel ill‐prepared for this role. Despite a multitude of programmes to support carer mental health, programmes that help carers feel better equipped to support a person with cancer are lacking. This study aimed to address this need by adapting an evidence‐based intervention to be suitable for carers of people with cancer.

**Methods:**

This study used an exploratory, qualitative design consisting of experienced‐based co‐design and an in‐depth stakeholder engagement strategy. An existing evidence‐based programme to promote resilience in the context of providing care was adapted for relevance to carers for people with cancer via two co‐design workshops with carers and healthcare professionals (*n* = 8). The resulting prototype programme was refined based on stakeholder consultations with staff and consumer members of cancer and carer support organisations across Australia (*n* = 16). Transcripts of the workshops, meetings and written feedback from carers were thematically analysed.

**Results:**

Major programme developments were guided by three themes that emerged from the co‐design workshops: ‘creating value for carers’, ‘multiple contributors to carer distress’ and ‘the need for flexible implementation’. Analysis of the stakeholder consultation data showed that the themes of ‘diversity in carer journeys’ and ‘creating impact for carers’ were key to further tailoring the programme for applicability to practice. An adapted programme called ‘iCanSupport’ resulted from the process, with key adaptations being more relevant case study scenarios for carers and greater flexibility in accessing and engaging with the intervention to accommodate a range of carer circumstances.

**Conclusion:**

Programmes to build skills for becoming a carer for someone with cancer are absent, yet they are desired by carers. Using co‐design provided a user‐centric approach to adapt an existing evidence‐based programme. Programme evaluation is required to determine the effectiveness of the co‐designed approach in improving carer preparedness among a range of cohorts.

**Patient or Public Contribution:**

Carers and consumers with lived experience and others involved in supporting consumers made valuable contributions to co‐designing and refining the programme in addition to providing ongoing guidance in the unfolding analysis and reporting of this research.

## 1 Introduction

The work of carers—often partners, parents, siblings, children and friends—is increasingly vital for both those who rely on them and the sustainability of cancer care [1]. Although not always acknowledged, carers are de facto members of the healthcare team [2, 3]. Carers' roles are complex and labour‐intensive, involving the provision of care in the home, community and healthcare settings such as hospitals [2, 4]. Carers often act as coordinators of care, navigators of health and welfare systems, decision‐makers and providers of clinical care (e.g., administering medications) [2, 4]. This can be highly challenging and may involve tasks such as ongoing monitoring of health status and detecting signs of sometimes rapid deterioration. The scope of this role has grown further with the recent expansion of community‐based and integrated models of care [4, 5].

Carers report significantly higher levels of anxiety than the general population; duties such as coordinating care have been attributed as a primary source of their distress [6]. Carers may experience a ‘dual burden’ [7], meaning they are simultaneously family members/friends experiencing distress due to their loved one's illness, which can include worry, guilt and a sense of over‐responsibility [8], while also attempting to manage caregiving‐related stress. Carers may also be attempting to balance caring for other family members and employment responsibilities and can face financial hardships among other impacts [9]. Carers have expressed a desire for interventions to help them prepare to navigate and manage challenges that arise [7].

There is recognition that cancer caregiving is multifaceted and that there are a range of aspects with which carers may benefit from supportive interventions to help address some of these challenges. These can include the following: health literacy enhancement programmes [10, 11, 12, 13], medical/clinical skills training [14] and programmes that promote carer psychosocial well‐being [15]. This study focuses on exploring psychosocial programmes that enhance resilience and help carers feel better prepared. Although the evidence concerning psychosocial interventions to support cancer carers is growing [16, 17, 18], there appears to be a lesser body of evidence specifically focused on the development and testing of programmes to help prepare carers for the journey ahead [19]. Preparedness is used to capture feelings of readiness and being equipped to take on a caring role [19]. Although preparedness may not be the priority in the immediate aftermath of a diagnosis, increasing feelings of preparedness during the cancer journey is associated with benefits such as reduced carer burden [20, 21]. Feeling ill‐prepared is a well‐established concern for carers, making it critical to address to enhance carers' well‐being [22]. Supportive interventions, including psychoeducational programmes, can be effective in increasing feeling able to cope and self‐competency to care [19]. Given the benefits associated with skills and resilience‐building interventions for managing distress associated with providing health care [23], programmes incorporating these elements are well‐positioned as preventative measures and help sustain well‐being in the course of providing care.

To address this need, an evidence‐based intervention specifically developed to promote resilience in the context of providing care was identified: ‘Reboot’ [23]. Developed to support healthcare professionals, Reboot is grounded in the Bi‐Dimensional Framework for resilience [24] and employs a cognitive behavioural therapy approach. Trialling of Reboot has shown increased resilience to feelings of stress associated with providing clinical care in a sample of students and qualified healthcare professionals (*n* = 66) [23], critical care nurses (*n* = 77) [25] and medical students (*n* = 115) [26]. These findings are encouraging, as resilience in carers is associated with carer burden, quality of life and other measures of well‐being [27, 28]. Reboot is promising but requires adaption to be suitable for carers. Preliminary findings from interviews with 20 carers confirmed the acceptability and potential relevance of a preparatory programme [7].

The views of carers and other stakeholders who would be involved in accessing and delivering the nominated programme are vital for successful adaptation and implementation. To our knowledge, there is little reporting of the involvement of these groups in the development of carer psychosocial interventions [29, 30]. This gap limits the sharing of learnings for future intervention development and poses the risk that interventions developed without carer guidance are not designed to account for their needs.

The primary aim of this study is to co‐design adaptations to an evidence‐based intervention specifically developed to increase resilience, enabling carers to feel ‘better equipped’ to provide care for people with cancer in Australia. The secondary aim of this study is to enhance understanding of the intervention adaptation process and identify development considerations.

## 2 Methods

### 2.1 Design

A qualitative study was undertaken of the process and outcomes of co‐designing adaptations to the Reboot programme. An experienced‐based co‐design approach was employed, which recognises the lived experience expertise of providing care [31, 32]. The process consisted of co‐design workshops to develop programme adaptions, stakeholder consultations to refine the revised programme and ongoing canvassing of feedback from participants to guide research team efforts.

### 2.2 Participants

Co‐design members were eligible who were:

- Carers: people aged 16 years and older with experience caring for a family member with cancer in Australia and who did not provide care in a professional capacity.
- Healthcare professionals with at least 12 months of experience of providing psychological support for people with cancer.

Stakeholder consultation group participants were:

- Staff members and consumer representatives (paid or voluntary) of cancer and carer support organisations.

### 2.3 Recruitment

The recruitment of all participants was facilitated by cancer support organisations and networks, in addition to snowball recruitment using clinical collaborators. Email invitations were disseminated via organisational distribution lists. The invitations encouraged anyone interested in participating to contact the project team for further discussion. We aimed to recruit no more than six co‐designers to ensure sufficient opportunity for everyone to contribute. Written, informed consent was obtained to collect data from the workshops, consultations and supporting communication in the design process. Reimbursement was offered to acknowledge carers' contributions and compensate their time as per guidelines [33].

### 2.4 Procedure

**Figure 1:**
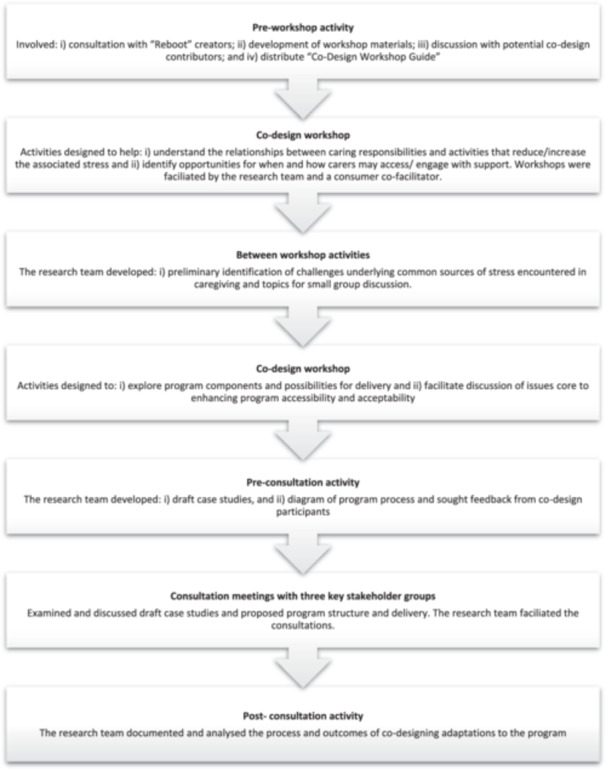
Overview of the iCanSupport programme development process.

An overview of the procedure is shown in Figure 1.

#### 2.4.1 Preparation

Discussion was undertaken with members of the team involved in creating Reboot to clarify the scope of adaptations possible while preserving the evidence‐based mechanisms. Drawing on a previously successful co‐design methodology [34, 35, 36], a consumer co‐facilitator contributed to developing the workshop materials and facilitating the sessions. Workshop contributors received a ‘Co‐design Workshop Guide’, which included details about what would be involved, and information about accessing the online videoconference platform (Zoom). Discussions were undertaken before and throughout the process with co‐designers to establish a process that suited their needs, confirm the scope of each person's role and to offer the support required to enable contributions.

#### 2.4.2 Co‐Design Workshops

Co‐design workshops aimed to:

- develop scenarios to be used as case studies within the programme so these are relevant to the day‐to‐day stressors experienced by carers; and
- enhance the accessibility of the programme for this population.

**Figure 2:**
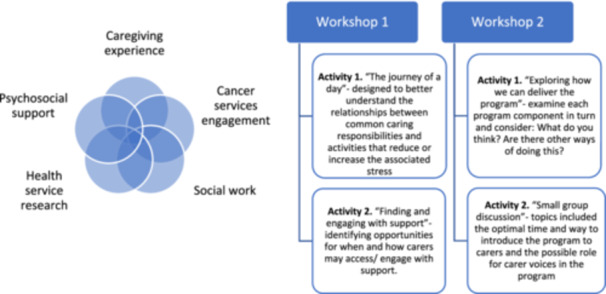
Co‐design workshop activities and contributor expertise.

Two online workshops were convened, each scheduled to be 2 h in length. The same group of participants were invited to contribute to both. Workshops comprise an introduction that encompasses a code of care for the group, study information and activities. See Figure 2 for activity details and the areas of contributor expertise.

Case study development began in Activity 1, where common and potentially stressful caring responsibilities were identified, and contributors considered how supported they felt in navigating these experiences. Researchers then analysed this discussion to uncover some common challenges underpinning these experiences, which were subsequently presented for discussion in the second workshop.

**Figure 3:**
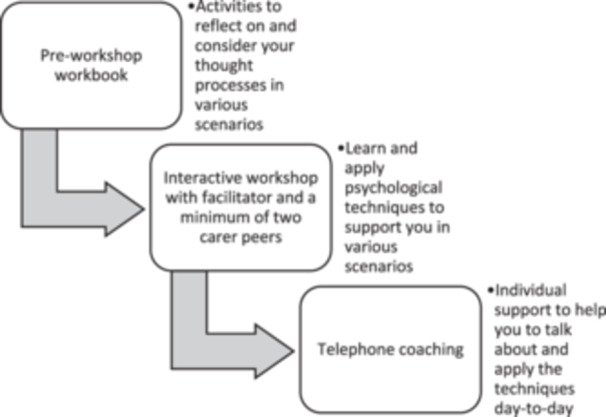
Original pre‐co‐design Reboot programme structure.

To remodel the programme for carers, opportunities for when and how carers may access and engage with support were explored in Activity 2 of the first workshop. The structure and components of the Reboot programme were examined (Figure 3). Original pre‐co‐design Reboot programme process) and possible adaptions in programme organisation and delivery that could enhance participation were discussed in the second workshop.

The research team then developed draft case study scenarios and a programme process flow diagram that was circulated to co‐designers for feedback. At points during the study, feedback was sought via email from co‐designers and stakeholders to guide the evolving understanding.

#### 2.4.3 Stakeholder Consultations

The perspectives of staff and consumers of cancer and carer support organisations were sought on the updated programme to refine its relevance and acceptability for carers. Consultations were conducted with three stakeholder groups via videoconference. The meetings were structured to include the following: an introduction, a study update and a discussion of the draft programme.

#### 2.4.4 Data Collection and Analysis

Workshops and consultations were audio recorded, and researchers made field notes. Written participant feedback was also collated. Field notes and participant feedback informed the development of the draft programme. Audio recordings were transcribed verbatim. These texts were read and re‐read to deepen familiarity with the data. Texts were coded and preliminary themes were constructed by one researcher [37]. Preliminary themes were refined via iterative engagement with the data set and team feedback. Themes were finalised by sharing these with contributors for further feedback and validation [38].

## 3 Results

Across the two co‐design workshops, three carers and two healthcare professionals (clinical psychologist and social worker) participated. One carer was unable to attend Workshop 1, and one healthcare professional did not participate in Workshop 2. Stakeholder consultations were undertaken with three organisations (see Table 1 for a summary of participants). Organisations were national and peak bodies that support carers, people diagnosed with cancer and their families. The bodies have consumer leadership and/or representation and work closely with other organisations in Australia and internationally.

**Table 1:** Summary of participants.

Co‐design workshops (total of 4 h)		Stakeholder consultations with three groups (total of 5 h)	
Carers	*n* = 3	Carers	*n* = 5
Healthcare professional	*n* = 2	Staff members/volunteers	*n* = 6
Consumer co‐facilitator	*n* = 1	Carers and staff members/volunteers	*n* = 2

### 3.1 Thematic Analytical Findings

The themes and subthemes resulting from the analysis of the co‐design workshops and stakeholder consultations are presented in Table 2.

**Table 2:** Overview of themes and subthemes.

Study component	Themes	Subthemes
Co‐design workshop findings: Adapting the programme for carers	1. Creating value for carers	
	2. Multiple contributors to carer distress	
	3. The need for flexible implementation	
Stakeholder consultation findings: Revising the programme for implementation	4. Diversity in carer journeys	
	5. Creating impact for carers	5.1. Availability to engage
		5.2. Creating a supportive environment
		5.3. Mitigating risk associated with participation

### 3.2 Co‐Design Workshop Findings: Adapting the Programme for Carers

#### 3.2.1 Theme 1: Creating Value for Carers

Carers may not view engaging with a programme for their own well‐being as a priority. It was suggested in the second workshop that clarifying the programme's focus on skills development and feelings of self‐competency, rather than offering support groups or counselling (which carers may be more familiar with), would help:

I would have fallen over myself looking back now I should have done this [program] but at the time I – …was spending time with what I had … how do you explain to people to go: ‘You know what? If things go bad, it can go bad really quick, and you'll look back on these phone calls and potentially will be really good for you.’

(Workshop 2: 848–853)

Discussion in the co‐design workshops explored whether framing the programme as training rather than support could better convey what the programme offers.

He was just getting through the treatment, and he would have accepted if I was going there from a training perspective.

(Workshop 2: 1082–1084)

[My preference] would be support … rather than training. So I'd prefer to hear: ‘Here's support for you’, and for me the messaging that I would have liked to have heard was: Here's a program to navigate you through this next period, whatever [way] you want to put it but how we're going to support you through the next period. There's an opportunity for training.

(Workshop 2: 1201)

Therefore, establishing what the programme offers carers and the associated benefits was critical to present a value proposition for carers.

#### 3.2.2 Theme 2: Multiple Contributors to Carer Distress

Discussion of responsibilities, activities and interactions encountered during care in the first workshop illuminated some challenges carers encountered. The first challenge was that managing oneself, others and the person being cared for simultaneously can create distress. Carers described having a range of responsibilities (e.g., direct clinical care) while attempting to manage their own well‐being and that of others. At times, this involved managing communication for family, friends and others:

Managing your emotions … especially when you've got another child in the house and when you knew things weren't going the way they should be and trying to keep it at the one level the whole time, is extremely difficult.

(Workshop 1: 234–238)

Providing emotional support and for [person being cared for] but also for others. You find yourself supporting other people as well and that links in with the other role which is actually often being the one who communicates with other family members and friends.

(Workshop 1: 266–269)

…if we're talking about parents, they definitely take the leading role in navigating, messaging out to the broader family, the extended family, the community….

(Workshop 1: 345–350)

Another challenge that emerged was that communicating with healthcare/services is complex and can create distress for carers. This included instances where the messages from healthcare professionals were not always easy to understand:

…communication with the healthcare professionals is hard because they talk to you in their language.

(Workshop 1: 596–600)

…maybe for some of them [health professionals] it probably felt like stating the bleeding obvious but sometimes you need someone to state the bleeding obvious. And I don't know whether there's sometimes an assumption that you would just know that this is what's happening. I'm sure it's difficult and I'm sure it's uncomfortable in all of that but sometimes you … just have to be able to tell it like it is.

(Workshop 1: 698–704)

Furthermore, the effectiveness of the channels for communication and the quality of communication varied, which was stressful for carers in need of advice:

[Oncologist] got back fairly promptly … we would sometimes contact … the clinic, they always answered the phone but whether it was a helpful conversation or not was not always the case. There were some elements of contacting his specialist that I would have to say worked well, some of the other contacts – and [health professional] also, some of the others not so.

(Workshop 1: 502–508)

The final challenge was that being in hospital was stressful, and compounded already stressful experiences, such as managing your own emotions while supporting others. In addition to the lack of privacy, managing food and sleep was described as particularly difficult while trying to provide comfort and care in hospital:

Managing emotions was very difficult especially when you're in a ward of four … you sit there, and everyone's got their own story, and everyone's got their own pain going on and I found it really difficult to manage that and what – not that there's an appropriate time … but you just didn't feel like you wanted to just ball your eyes out which deep down you wanted to but you're like hang on, we've got other families here.

(Workshop 1: 643–648)

food was really difficult … I've got sleep but again not just for [person being cared for] but, for me as well, so [person being cared for] be up all night spewing or whatever was going on in [their] little body and I was sleeping on this crappy pull‐out bed … I wouldn't get any sleep and then day time doctors would be in, everyone's coming in and you'd lose your marbles to be honest because you just were operating on such a small amount of sleep.

(Workshop 1: 628–635)

The identification and interpretation of these insights were confirmed with carers in the second workshop. The case studies drafted drew on these challenges (Table 3).

**Table 3:** Overview of draft case study scenarios.

Case study	Relationship	Setting	Key challenges
1. Danni and Mo	Caring for a partner	Home setting	Managing communication in the family
			Balancing responsibilities and commitments
2. Jo and Clara	Caring for a child	Hospital outpatient setting	Preparing for appointments
			Keeping track of everything
			Organising the family and household
3. Jai and Edwin	Caring for a sibling	Inpatient hospital setting	Sole carer and source of emotional support
			Monitoring care and engaging with healthcare professionals

*Note:* Please see Supporting Information: S1 for a case study example.

#### 3.2.3 Theme 3: The Need Flexible Implementation

There was an acknowledgement among the co‐design group of the limited time carers have and that committing to plans can be difficult. Given this, contributors suggested that ad hoc opportunities for programme engagement or options to negotiate times/dates to suit could help:

…there's almost the need to be a little bit flexible because what if I was planning to do that at one o'clock today, but it's gone a bit pear shaped… – but I'm wondering whether it's something that could be negotiable. That once someone engages with the program then you negotiate as best you can with the psychologist and the peer around when we might go online together or when we might connect via a group chat….

(Workshop 2: 719–726)

…it could be helpful to think about those opportunistic ways like audio, podcast, things that people can dip into and those intermediate times between things.

(Workshop 1: 853–857)

Furthermore, contributors proposed that dividing sessions/content into smaller ‘chunks’, rather than lengthier commitments, makes it more feasible for carers:

if it is possible for what you would normally achieve in the workshop to be broken down into smaller chunks, I think that would seem more do‐able … if it appeared that as a carer you needed to find two hours to engage with this, I think that would be or would feel prohibitive.

(Workshop 2: 695–700)

Offering digital and online options to engage was thought by contributors to also help align with the opportunities that carers have to participate, including potentially facilitating ad hoc engagement:

…it would need to be something that you can access either through an app or online in some way and then you can do it in your own time.

(Workshop 2: 507–509)

…face‐to‐face is great but I think it would be easier for people to engage if they didn't actually have to get themselves to an appointment. That they can do it over the phone or online I think would be, they would be more likely to feel like it was something that they could actually engage with.

(Workshop 2: 732–736)

**Figure 4:**
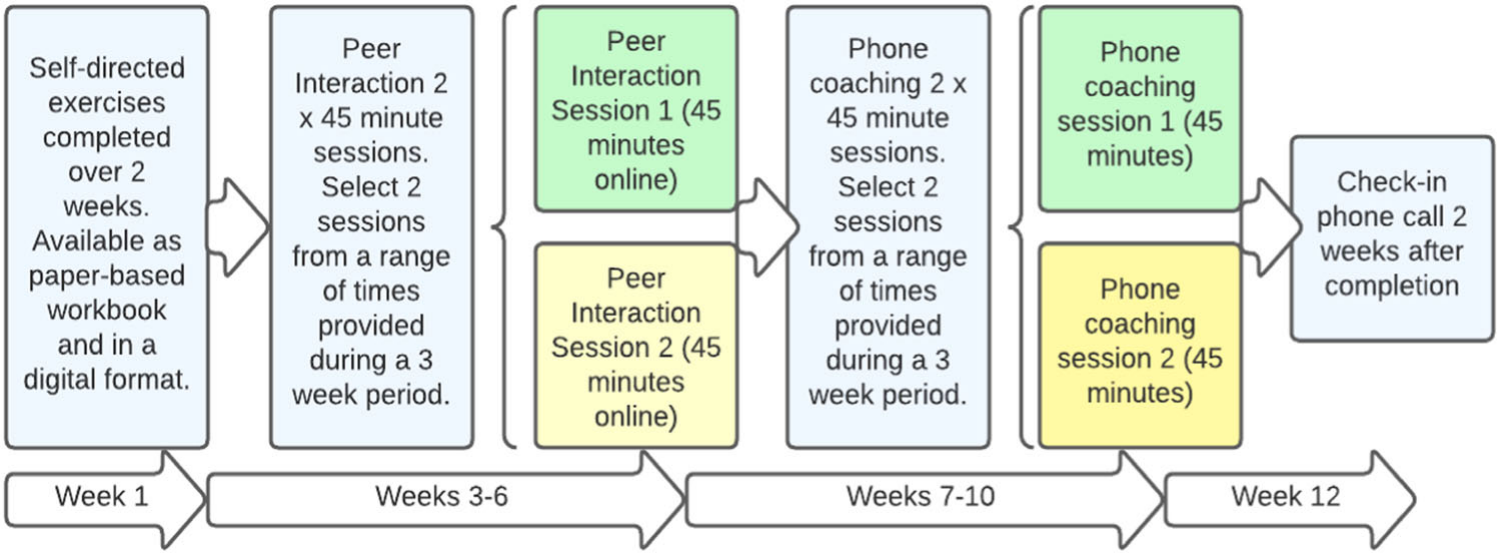
Revised draft programme structure.

These discussions resulted in a revised draft programme shown in Figure 4.

### 3.3 Stakeholder Consultation Findings: Revising the Programme for Implementation

#### 3.3.1 Theme 4: Diversity in Carer Journeys

Stakeholder discussions highlighted the diversity in carer journeys and prompted consideration of whether the programme should be tailored for a specific carer population and whether programme content can be equally relevant across circumstances. Although, in many instances, the underlying challenges of the draft case studies (e.g., responsibility for managing communication) were relatable, the scenario or certain details were not always relevant to different populations, treatments and prognoses. This point was captured in a consultation:

I can relate to the case study, … as a former carer of a parent with cancer … it took me right back there around having to be the guide, between having to be the communicator … keeping the other family members informed as soon as I got home, trying to run a family.

(Consultation 2: 466–467)

You wouldn't be going to anywhere else to get a blood test [referring to the case study] … the reality is, you're doing everything in hospital most of the time, so it doesn't even feel that relatable to me.

(Consultation 2: 638–641)

Differences in experiences were also reflected upon in their significance for organising peer interaction sessions:

Sometimes it's quite difficult for a parent who's got a child on stage four treatment to be in a conversation with a parent who's got a stage one or two child on treatment, because the treatment programs are totally opposite ends of really … the spectrum…, diagnosis is really different … it probably would be better to put relapsed parents separately.

(Consultation 2: 325–332)

*Facilitator*: …what are your thoughts on being part of a group with people whose children, who may not even have a child. It may be that it's somebody who has a spouse that has a diagnosis of cancer or someone caring for another family member with a different cancer type or a child with a different cancer type…. Would you think that that would still be of value, or perhaps be a whole different way of approaching things?

*Speaker*: with the [peer support sessions] I do … there's a range of different [carers] and sometimes they're teenagers rather than toddlers … so there are some differences then that they talk about … if you're talking about the skills that could apply to all of them, and I'm also a member of [support group meeting] that they do, and so in that there are some wives that their husbands have cancer … But we still yeah, there are still always common themes that they bring up.

(Consultation 1: 342–350)

There were several suggestions for additional case studies, and thought was given to whether offering a library of case studies that carers would choose from might be valuable:

[re: case study suggestion] … an adult child caring for an older parent. So, when I worked in hospital we saw that quite often where the older their parent is diagnosed with cancer, and that adult child is looking after their parent with cancer.

(Consultation 3: 251–253)

[re: library] Say you pick a story where they've got like three questions: I'm a parent, and I put it, child, or whatever, and then they go here is the top three recommended case studies and then see here for more.

(Consultation 3: 254–255)

Offering a wider range of case studies to reflect an array of circumstances provides a possible way to respond to some of the challenges identified.

#### 3.3.2 Theme 5: Creating Impact for Carers

For a programme to have the intended impact, the implementation strategy ought to factor in the availability of carers to adhere to programme processes, which is described in relation to the subthemes: availability to engage, creating a supportive environment and mitigating risk associated with participation.

##### 3.3.2.1 Subtheme 5.1: Availability to Engage

Stakeholders shared the view initially proposed by co‐designers that dividing sessions/content could enhance carers opportunities to engage in the programme and explored further opportunities to ‘chunk’ workbook content:

…putting it in a digital format lends itself to that, to that snappiness … with those short bits of information … they're in the hospital, they're on their phones, even having like an app or something they can easily access.

(Consultation 3: 91–92)

Stakeholders considered whether the programme required a sequential approach and the possible associated impacts on engagement. Some of those consulted perceived that if one component is a pre‐requisite to another, this could be barrier to ongoing programme engagement:

Completing the workbook in the early stages [is] difficult as it might be an overwhelming time and if you don't complete the pre‐book, are you able to participate in the session?

(Consultation 3: 119)

Scheduling arrangements for synchronous interactions were also a central topic. Consistent with discussion in the co‐design sessions, stakeholders identified flexibility as essential for carers and advanced ideas about how possible times/dates could be selected with ample notice and, where needed, rescheduled:

[re: booking sessions] in my experience, [it is] not ample time for a carer, or 3 weeks is very often the minimum amount of time that it takes to arrange something. And then there needs to be some sort of flexibility, and then, like it was said before outside office hours, outside work hours.

(Consultation 2: 233–234)

[re: booking sessions] … keep in mind if they book something in, there's a high chance that they may not be able to show up.

(Consultation 3: 153–156)

One suggestion that merits further reflection is that programme participants may wish to work with the same facilitator throughout:

Some of that value comes from the continuity of having the same person all the way through. So if I'm going to have a phone coaching session, I would like that to be with the person that I had who led the interaction or otherwise you kind of end up starting from scratch again, don't you?

(Consultation 3: 133–134)

However, it may be challenging to reconcile the preference for consistency with the need for flexibility described previously.

##### 3.3.2.2 Subtheme 5.2: Creating a Supportive Environment

The need to ensure comfort and enhance support for those engaging in the programme was discussed in the stakeholder consultations. There was an acknowledgement that carers will likely encounter this programme at a distressing time, which shapes how they may wish to engage with it. Through this lens, the circumstances in which the first activity, the workbook, is completed and the content of the case studies was reflected upon by all the stakeholder groups:

I like to be making notes. I guess that's a bit of an anxious thing as well, which I am more anxious now that my child has got cancer, so I find it really helpful to have this physical workbook and then I also keep it in his hospital bag, so when he goes for scans, I've got some time then, and I don't usually have time at home when he's awake. So then I have this with me to do some of the extra activities. I haven't opened the PDF.

(Stakeholder Consultation 1: lines 255–259)

[re: case studies] if you've had a difficult day, or if you have a difficult relationship to this, it might you know, not be the best, most supportive thing for you to read and I think I don't know if there's a way to do like it's, you know, probably asking the impossible. But is there a way to do it like that is more strength based or situational.

(Stakeholder Consultation 2: lines 454–456)

[re: case studies] it can be very raw for people … so it's sort of opened up this can of worms, so to speak. So where do I go now? Do I have to wait 2 weeks, or whatever to talk about.

(Stakeholder Consultation 2: lines 470–471)

[re: case studies] I just kind of wonder whether you're introducing this scenario at the very start of somebody's cancer journey, whether that's actually going to make them, they're going to read that and they're going to go, this is really really awful, isn't it?

(Stakeholder Consultation 3: lines 198–200)

The fact that the workbook is proposed as the first component a participant would encounter is significant. Regardless of the activity, it may be helpful to include an orientation session that can provide details of support services. The way in which this programme would be situated in relation to complementary support services was reflected upon in the stakeholder groups, as carers may not necessarily have the full support they need. Stakeholders encouraged the integration of opportunities to connect participants with relevant support services in programme planning:

the other thing that I was thinking is referral pathways out of that [program] … I understand that the program aims to develop skills. Skills, aren't necessarily the only thing that's a problem. So, it would be good, I think, if there were certain points just check ins about whether other supports are needed.

(Consultation 2: 348–353)

how [do] you plan to manage the transition post program? … but they've opened up through this process because they thought it was more skills based, they've done their coaching session with the psychologists or social, or whoever it is and then, what, programs done?

(Consultation 3: 137–139)

##### 3.3.2.3 Subtheme 5.3. Mitigating Risk Associated With Participation

To enhance programme accessibility, participants suggested that aspects such as the language, compatibility with screen readers and user‐friendliness needed to be accounted for during programme design:

I'm just thinking about accessibility in terms of screen readers or translation as well, so I know that if it's in a word document, my screen readers can … it will be accessible that way.

(Consultation 2: 138–139)

content can come across a bit academic … it's using that really plain English that's understandable for the reader.

(Consultation 3: 224)

If a carer is working on an exercise on the digital format, can they pick up where they left off?

(Consultation 2: 125)

A simple and easy‐to‐navigate booking system was considered essential for carers to minimise additional burden resulting from engaging in the programme:

to book in those individual sessions, just make it a super easy … and people can change it if they need to, so it reduces the workload. It's probably a bit of an investment up front, but if you can get a very nice, easy booking system, it makes our lives easier.

(Consultation 1: 512–513)

Furthermore, in agreement with co‐design contributors, stakeholders felt providing options to facilitate different preferences (e.g., phone/video call) could promote accessibility:

I kind of like the tangible paper copy but I guess everybody's different … it's useful to have an online form or a tool, or even an app that people who are very digitally minded.

(Consultation 1: 269–272)

[re: case studies] I found that text quite heavy just to read and to look at it. And actually, I started glazing over the words because it was just so much … and whether there could be some little comic strip or video animation that could go alongside to help.

(Consultation 3: 187–188)

with phone coaching, would you give the option of a zoom call or online.

(Consultation 1: 504–505)

Stakeholders identified several steps to mitigate the risk that programme participation would contribute to, rather than reduce, the carer burden. Suggestions included reminder texts and email summaries to support ongoing programme engagement:

I really appreciate the emails I get from the [organisation] social workers after therapy video calls we have because they sort of just do a little bit of a minutes [sic]. So they just do like some headings of some topics that will brought it off [sic] … Yeah, one of those things that helps with learning just to recap things and seeing it in the in an email.

(Consultation 1: 487)

I like email, but I love getting a text reminder like that morning.

(Consultation 1: 503)

**Figure 5:**
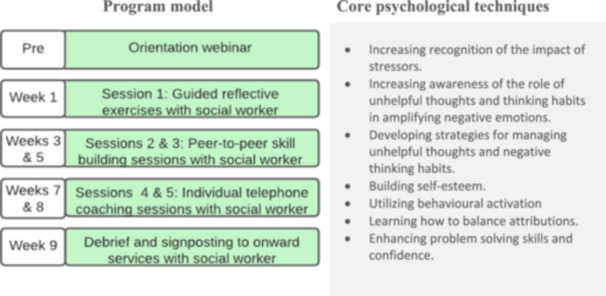
Finalised iCanSupport programme process and techniques.

Possible refinements that were identified as a result of the consultations were shared with the design group by email for feedback to finalise the newly adapted iCanSupport programme (Figure 5). The workbook component with the reflective exercises is now proposed as a guided activity with the support of a facilitator. The programme now also includes an orientation webinar (offered live or as a recording) and a debriefing session, which further embeds opportunities for referral to support as needed.

## 4 Discussion

A primary aim of this study was to co‐design adaptations to a programme for carers of people with cancer in Australia. Analysis of the co‐design workshops and feedback captured three themes that drove the changes to the programme needed to suit carer populations: ‘creating value for carers’, ‘multiple contributors to carer distress’ and ‘the need for flexible implementation’. Subsequent consultation with key stakeholders provided guidance about how the programme may best be refined, oriented by the themes of: ‘diversity in carer journeys’ and ‘creating impact for carers’. The newly adapted iCanSupport programme features two key differences: carer case study scenarios and a revised model of the structure and delivery of the intervention. Feedback from stakeholder consultations also offered possibilities for additional case studies and programme delivery refinement options.

Given that the programme is designed to help carers manage distress that may arise in the course of providing care, this may be understood in terms of building skills in the process of becoming a carer. This perspective has been adopted elsewhere with programmes focusing on providing carers with practical and emotional skills, particularly in the context of palliative and end‐of‐life care [19]. Framing the programme in this way may help better convey the nature and intended benefits of the intervention.

Ensuring that the focus and targeted outcomes of the programme are communicated (i.e., skills and resilience building) and distinguished from supportive programmes (e.g., counselling) that may be more commonly offered in this area, helps establish expectations for prospective participants and will be informative for clinicians seeking to identify relevant programmes for carers. As was raised in the stakeholder consultations, the programme ought to be well‐connected with supportive services that carers may find beneficial (e.g., support for financial management). Furthermore, there are opportunities to link this programme with others which may bolster support for carers in tackling some of the challenges they report encountering, such as health literacy programmes concerning healthcare communication, health system navigation and identifying and understanding health information [12, 13]. iCanSupport contains features that have been positively viewed by carers in previous research, such as the opportunity for peer interaction combined with facilitator involvement and offering online engagement options; however, there are also a range of considerations needed when seeking to put such programmes into practice [39, 40]. Furthermore, this process allowed for consideration of some aspects of sustainability, including the identification of social work professionals as a suitable cohort to deliver the programme; an approach successfully deployed in several programmes offered by the study partner organisations. The findings give rise to several considerations for implementation that may be explored in further work. For example, stakeholder consultations indicated that in programme implementation planning, strategies such as mapping referral pathways to these services would help embed this programme in existing oncology service structures and facilitate greater access to holistic care.

Similar to other co‐designed adaptions to programmes [17], the required adaptations to iCanSupport would not have been possible to surface without in‐depth reflection with people who have lived experience of caring and supporting carers. Revisions in the development and tailoring of programmes as a result of the contributions of these key stakeholders are consistent with intervention development efforts reported elsewhere [17, 41]. The current study illuminates the process of using co‐design to adapt an intervention and provides guidance for those similarly interested in partnering with carers and other stakeholders in programme development. Programme implementation, including concerning issues of sustainability will be guided and shaped by ongoing consultation with stakeholders with further revisions to be made as needed.

As noted elsewhere, the lack of reporting of co‐design and other collaboration with carers in the development of interventions indicates an under‐utilisation of the expertise vital to the success of such programmes [15, 29, 40]. To date, progress has been hindered by a lack of attention to preparatory programmes in the literature concerning psychosocial interventions for cancer caregivers [19]. This body of work also includes reporting interventions that are dyadic (e.g., patient–caregiver) or have been developed for patients and extended to carers. A recommendation is that the expertise of carers and those who support them are central in initiatives to design and implement research and policies, which aim to benefit them. Without a central, primary focus on carer needs to guide programme development, there is a risk that the needs of this group may not be met.

## 5 Limitations

The diversity of carers' journeys, their engagement preferences (including with online/digital technology), health literacy and the opportunities they have to participate emerged as points for reflection in the development process. Recognising that it is not possible for the totality of differing circumstances and journeys to be represented in this process, an implication of this research is the need, going forward, to consider any additional barriers or needs that specific carer groups may have for participating in the programme and account for this in implementation planning.

## 6 Conclusion

This study contributes to efforts to best equip carers to navigate the stress arising in the course of providing care. iCanSupport addresses a gap in the current programmes available for carers. The use of co‐design is a key aspect that has enabled us to consider the content and implementation support required. The next step is to evaluate the programme with carers among people with cancer, inclusive of clinical outcomes, and then consider applications to further carer cohorts.

## Author Contributions

**Bróna Nic Giolla Easpaig:** formal analysis, data curation, project administration, writing–review and editing, writing–original draft, funding acquisition, investigation, methodology. **Bronwyn Newman:** investigation, funding acquisition, writing–original draft, methodology, writing–review and editing, formal analysis, project administration, data curation. **Judith Johnson:** conceptualisation, investigation, writing–review and editing, methodology, formal analysis, data curation, project administration. **Ursula M. Sansom‐Daly:** investigation, writing–original draft, writing–review and editing, methodology, formal analysis, data curation, project administration. **Lucy Jones:** investigation, writing–review and editing, methodology, formal analysis, project administration, data curation. **Lukas Hofstätter:** investigation, writing–review and editing, methodology, formal analysis, project administration, data curation. **Eden G. Robertson:** investigation, writing–review and editing, methodology, formal analysis, project administration, data curation. **Reema Harrison:** conceptualisation, investigation, funding acquisition, writing–review and editing, methodology, formal analysis, project administration, data curation.

## Ethics Statement

Ethical approval was granted by a university Human Research Ethics Committee (reference no: H22006).

## Conflicts of Interest

The authors declare no conflicts of interest.

## Supporting information

Supporting information.

## Data Availability

The data presented in this manuscript cannot be made available as per the conditions of ethical approval.
