# Supplementary material for: Adapting a Preparatory Skills‐Building Programme for Carers of People With Cancer Through Co‐Design: The iCanSupport Project
Source: Health Expect. 2024 Oct 17;27(5):e70061. doi: 10.1111/hex.70061 (PMC11483551; doi:10.1111/hex.70061)
Supplement: Supplementary file 1 — Supporting information. [file HEX-27-e70061-s001.docx]

**Supplementary File 1.** Example Case Study

| Case Study 1: Danni & Mo  **Population:** Carer for partner  **Location:** Home  *Danni is caring for her partner Mo who has recently been diagnosed with bowel cancer and undergone surgery. Mo has chemo fortnightly, regular medical follow up appointments and takes multiple medications daily. At first Mo could manage his own medication but has found it difficult to manage on his own as the chemo sessions progress. Danni feels responsible for making sure that all the correct medications are taken at the right time, and that nothing is forgotten. Since his surgery, Mo can eat a modified diet, which takes careful preparation and is not suitable as a family meal.*  *Danni supports Mo alongside many ongoing responsibilities - she continues to work part time, support her aging parents and has a school aged family living at home. Mo’s sister Ali calls by daily to assist with the washing and after school tasks. At each visit Ali expects updates on Mo’s diagnosis, progress and day to day care. On the days where Mo attends appointments Ali collects the children from school and is waiting to hear how things went when Danni and Mo arrive home. Mo and the children are often impacted by Ali’s questions and her level of distress and concerned about who else Ali tells things to. The family and Mo rely on Danni to filter information and communicate with Ali when she visits. Danni feels conflicted- not sure how the household would function without Ali, concerned for Ali in her distress, yet responsible for protecting Mo and the children from Ali’s questions and interactions, and determined to have energy for day-to-day life with Mo - as he is her priority.* |
| --- |
